# Retropharyngeale Tendinitis: Seltene Differenzialdiagnose bei akuten Nackenschmerzen

**DOI:** 10.1007/s00113-021-01138-9

**Published:** 2022-01-13

**Authors:** Olivia Mair, Jens Gempt, Martin Renz, Peter Biberthaler, Marc Hanschen

**Affiliations:** 1 Fakultät für Medizin, Klinikum rechts der Isar, Klinik und Poliklinik für Unfallchirurgie, Technische Universität München, Ismaninger Str 22, 81675 München, Deutschland; 2 Fakultät für Medizin, Klinikum rechts der Isar, Klinik und Poliklinik für Neurochirurgie, Technische Universität München, München, Deutschland; 3 Fakultät für Medizin, Klinikum rechts der Isar, Institut für diagnostische und interventionelle Radiologie, Technische Universität München, München, Deutschland

**Keywords:** Odynophagie, Prävertebrale Tendinitis, Akute Nackenschmerzen, Akute kalzifizierende Tendinitis des M. longus colli, Fallpräsentation, Odynophagia, Prevertebral tendinitis, Acute neck pain, Acute calcific tendinitis of the longus colli muscle, Case report

## Abstract

Atraumatische Nackenschmerzen sind ein häufiger Vorstellungsgrund für Patienten in der Notaufnahme. Für die behandelnden Ärzte ist dabei der Ausschluss möglicherweise lebensbedrohlicher Erkrankungen, wie Spondylodiszitis, retropharyngealer Abszess oder Meningitis, oft eine große Herausforderung. In dieser Kasuistik wird der seltene Fall der retropharyngealen Tendinitis vorgestellt, einer wenig bekannten und dadurch sicherlich unterdiagnostizierten Entität. Sie ist charakterisiert durch einen stark reduzierten Bewegungsumfang der HWS, erhöhte Infektparameter und einen pathognomonischen MRT-Befund.

Zusätzlich zur Fallpräsentation soll im Rahmen eines kurzen Reviews die Charakteristik der retropharyngealen Tendinitis noch genauer dargestellt werden. So ist es das Ziel dieser Arbeit, die behandelnden Ärzte für dieses Krankheitsbild zu sensibilisieren, um in Zukunft falsche bzw. unnötige Therapien zu vermeiden.

## Abstract (en)

Atraumatic neck pain is a frequent reason for patients to attend the emergency department (ED). It remains a challenge for the treating physician to rule out possible life-threatening causes, such as a retropharyngeal abscess, meningitis or septic spondylodiscitis. Herein, we report the rare case of a patient suffering from retropharyngeal tendinitis. This rare cause of atraumatic neck pain is characterized by a significant decrease in the range of motion of the cervical spine, elevated infection parameters and pathognomonic MRI findings. In addition to presenting the rare case of retropharyngeal tendinitis, we also review selected previous case reports and thereby not only raise awareness for this rare disease but also suggest the best practice for treatment.

## Falldarstellung

### Anamnese

Ein 34-jähriger Patient stellte sich über die chirurgische Notaufnahme unseres überregionalen Traumazentrums mit akuten Nackenschmerzen, Kopfschmerzen und Schluckbeschwerden vor. Der Patient berichtete, dass die Schmerzen vor etwa 3 Tagen plötzlich ohne auslösendes Trauma begonnen hätten, woraufhin die initiale Vorstellung in der Notaufnahme eines naheliegenden Krankenhauses erfolgte. Dort sei ein akutes Zervikalsyndrom diagnostiziert worden und der Patient mit Schmerzmitteln (Metamizol) und einer weichen Halskrause entlassen worden. Da sich die Beschwerden im Verlauf nicht gebessert hätten, erfolgte die erneute ärztliche Vorstellung bei uns.

### Befund

Klinisch zeigte sich ein ca. 185 cm großer, 80 kg schwerer, kardiopulmonal stabiler, 4‑fach orientierter männlicher Patient ohne Vorerkrankungen oder Eigenmedikation.

Der Bewegungsumfang in der Halswirbelsäule war in allen Ebenen stark eingeschränkt und stark schmerzhaft (VAS 9/10). Bei Palpation war kein paravertebraler Muskelhartspan tastbar oder Schmerz auf Druck auslösbar. Die periphere Durchblutung, Sensibilität und Motorik der oberen Extremitäten waren unauffällig. Zusätzlich berichtete der Patient von Schmerzen beim Schlucken und nuchalen Kopfschmerzen. Die Körpertemperatur lag bei 36,7 °C.

Laborchemisch zeigten sich erhöhte Entzündungsparameter (Leukozyten: 11,2 Gpt/l, CRP: 8,0 mg/dl (Referenzbereich: <0,5 mg/dl)) bei sonst unauffälligem Labor. Ein PCR-Test für SARS-CoV‑2, Influenza-A/B- und RS-Virus war negativ.

Im Rahmen der konsiliarischen Vorstellung bei den Kollegen der Hals-, Nasen-, Ohrenheilkunde konnte kein Auslöser für die Dysphagie gefunden werden.

### Bildgebung

**Figure 1:**
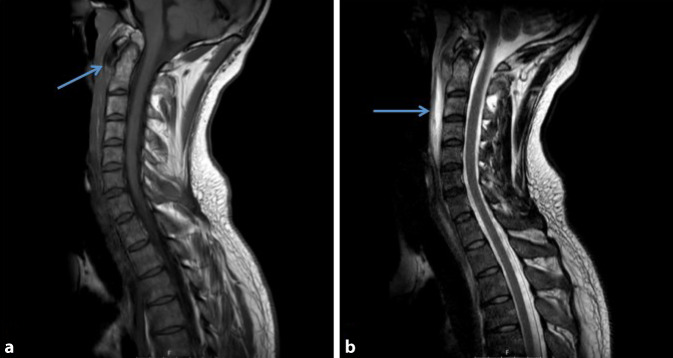

Aufgrund des Symptomenkomplexes aus Odynophagie, erhöhten Infektparametern und ausgeprägten Schmerzen in der Halswirbelsäule wurde zum Ausschluss einer Spondylodiszitis, eines retropharyngealen Abszesses und eines Bandscheibenprolaps eine notfallmäßige MRT des Halses *mit entsprechenden Kontrastmittelsequenzen* durchgeführt. Hier zeigten sich eine prävertebrale Flüssigkeitsansammlung ventral von C1–C5 ohne Verbindung zur HWS oder zum Retropharyngealraum und eine Kalzifikation ventral des Dens axis (Abb. 1a, b).

**Figure 2:**
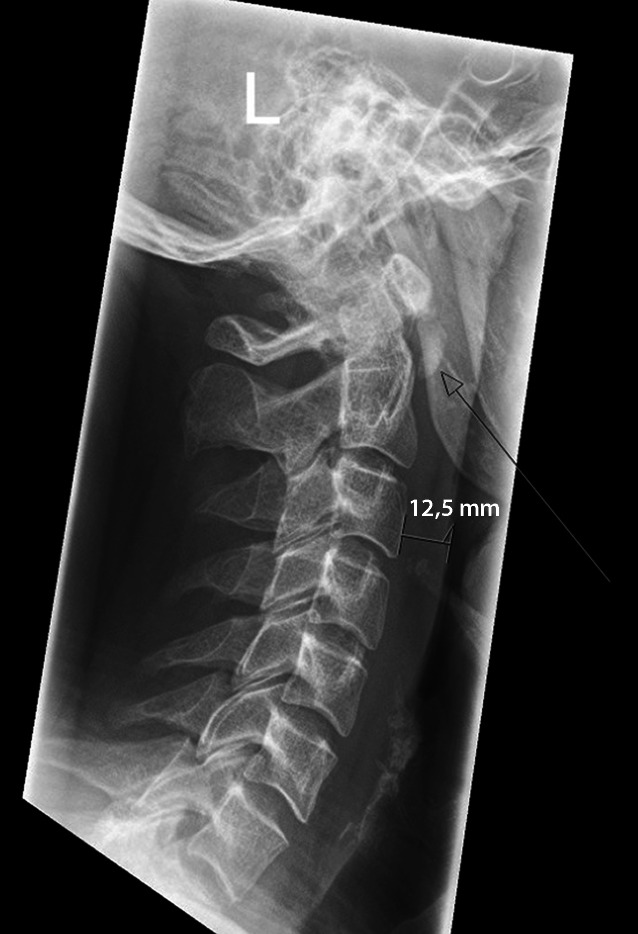

Um die Diagnose zu sichern, wurde durch den befundenden Radiologen ein laterales Röntgen der HWS angeordnet, wo sich die prävertebrale Weichteilschwellung und die Kalzifikation abermals bestätigten (Abb. 2) und so die Diagnose gesichert war.

### Therapie und Verlauf

Dem Patienten wurde eine Hochdosistherapie von NSAR (Diclofenac 75 mg p.o. 1‑0-1 + Paracetamol/Codein 500/30 mg p.o. 1‑1-1-1) in Kombination mit einem Protonenpumpeninhibitor (Pantoprazol 40 mg p.o. 1‑0-0) verordnet. Die weiche Halskrause wurde abgelegt und der Patient nach Hause entlassen.

Bei der klinischen Verlaufskontrolle 5 Tage später zeigte sich der Patient deutlich schmerzreduziert (VAS 4/10), bei fast normalem Bewegungsumfang in der HWS. Der Patient berichtete, dass schon nach 2 Tagen die Schmerzen deutlich regredient gewesen seien und die Schluckbeschwerden nach 3 Tagen vollständig sistierten.

## Diskussion

Die retropharyngeale Tendinitis, auch akut kalzifizierende Tendinitis des M. longus colli genannt, ist eine benigne und seltene Ursache für akute Nackenschmerzen. Etwa 120 Fälle wurden bisher in der Literatur berichtet, und die Inzidenz wird auf ca. 0,5/100.000 Personen pro Jahr geschätzt [1, 2].

Verursacht wird die retropharyngeale Tendinitis durch Ablagerung von Hydroxyapatit in den oberen Zügeln des M. longus colli.

„Hydroxyapatite deposition disease“ (HADD) ist eine gut bekannte Entität und kann theoretisch in jeder Sehne des Körpers vorkommen. Am häufigsten sind hiervon jedoch die Schulter und die Hüfte betroffen [3]. Die abgelagerten Hydroxyapatitkristalle verursachen dabei eine Entzündungsreaktion im Körper, was zu einem periinflammatorischen Ödem führt und auch von systemischen Reaktionen (z. B. Fieber, Leukozytose, etc.) begleitet sein kann [4]. Die genaue Ätiologie der HADD ist jedoch nicht abschließend belegt. So stützen einige Autoren die Theorie der zellvermittelten Entzündungsreaktion, wohingegen andere Autoren eher von auslösenden Mikrotraumen, degenerativen Prozessen oder genetischer Prädisposition ausgehen [3].

Der Symptomenkomplex aus atraumatischen, starken Nackenschmerzen, deutlich reduzierter RoM in der HWS, Meningismus und Odynophagie bei leicht erhöhten Infektparametern ist typisch für die retropharyngeale Tendinitis.

Es gilt, mittels gezielter Diagnostik durch sensibilisiertes Personal einerseits Fehldiagnosen und hierdurch bedingte Überbehandlung zu vermeiden, andererseits die schwerwiegenden Differenzialdiagnosen mit ähnlicher Symptomatik, insbesondere die septische Spondylodiszitis, den retropharyngealen Abszess und die Meningitis, sicher auszuschließen.

Durch einen erfahrenen Radiologen kann die Diagnose der retropharyngealen Tendinitis auch schon anhand eines einfachen lateralen Röntgenbildes der HWS sichergestellt werden (Abb. 2). Auch in der Computertomographie mit Kontrastmittel werden die charakteristischen Befunde, insbesondere die prävertebrale Flüssigkeitsansammlung und das prävertebrale Kalkdepot, auf Höhe der oberen HWS gut sichtbar.

Nichtsdestotrotz ist die MRT-Diagnostik des Halses und der HWS mit entsprechenden Kontrastmittelsequenzen der Goldstandard für die Diagnostik der retropharyngealen Tendinitis.

Hier sind zum einen die oben genannten pathognomonischen Befunde am deutlichsten erkennbar, zeitgleich können aber auch potenziell lebensbedrohliche Differenzialdiagnosen sicher ausgeschlossen werden. Um die diffuse, nichtabgekapselte prävertebrale Flüssigkeitskollektion der retropharyngealen Tendinitis zeigt sich, im Gegensatz zu einem Abszess, keine Kontrastmittelanreicherung in den T2-gewichteten MRT Sequenzen. Eine fachspezifische HNO-ärztliche Untersuchung sollte zum sicheren Ausschluss eines Retropharyngealabszesses zusätzlich erfolgen. Zudem hat die Flüssigkeitskollektion keine Verbindung zur HWS, wodurch auch eine septische Spondylodiszitis in dieser Konstellation als sicher ausgeschlossen gilt [2, 5]. Gleichzeitig können mittels MRT auch ein Bandscheibenprolaps, eine Spinalkanalstenose etc. differenzialdiagnostisch evaluiert werden. Zudem wird in den T1-gewichteten Sequenzen des MRT das Kalkdepot eindeutig sichtbar.

Außerhalb der regulären Dienstzeiten ist eine notfallmäßige MRT besonders in peripheren Krankenhäusern häufig nicht verfügbar. Somit kann bei Unsicherheit des behandelnden Arztes oder notfallmäßig nur verfügbarer CT-Bildgebung insbesondere im Hinblick auf mögliche medikolegale Aspekte ggf. die stationäre Aufnahme zur engmaschigen klinischen Überwachung des Patienten empfehlenswert sein. In Zusammenschau der verfügbaren Befunde (konventionelles Röntgen, CT, Labor) sind auch bei stationärer Aufnahme des Patienten prophylaktische Antibiotikagaben, Feinnadelpunktionen, Lumbalpunktionen oder gar eine operative Exploration nicht nötig und sollten somit auch dringend vermieden werden. Die Abnahme von Blutkulturen ist bei vorliegender Verdachtsdiagnose der retropharyngealen Tendinitis zwar nicht notwendig, kann aber die Diagnose der retropharyngealen Tendinitis hinzukommend sichern [2, 6, 7].

Zusammenfassend muss erwähnt sein, dass die sichere Diagnosestellung der retropharyngealen Tendinitis fundierte Kenntnisse dieser Entität durch den behandelnden Arzt voraussetzt.

Therapieoptionen sind insgesamt limitiert. In der Literatur (Tab. 1) empfohlen wird die hochdosierte Therapie mit NSAR, welche ggf. durch orale Kortikosteroideinnahme ergänzt werden kann. Ein Unterschied in der Schmerz- und Symptomfreiheit konnte jedoch nicht nachgewiesen werden [5, 7, 8].

**Table 1:** 

	Publikationstyp	Alter (in Jahren)	Geschlecht	Hauptsymptome	Labor	CT-Befund	MRT-Befund	Therapie	Tage bis Genesung
*Dieser Case report: Mair et al.*	Case report	34	m	Starke Nackenschmerzen, reduzierte RoM, Kopfschmerzen, Odynophagie	WBC: 11,2 Gpt/l CRP 8,0 mg/dl	n. a.	Prävertebrale Flüssigkeitsansammlung und Kalzifikation	Diclofenac 75 mg 2‑mal/Tag + Paracetamol/Codein 500/30 mg 4‑mal/Tag	7
*Zipfel et al. *[8]	Case report	34	m	Nackenschmerzen, reduzierte RoM	WBC: 11,8 Gpt/l CRP: 4,1 mg/dl	Prävertebrale Flüssigkeitskollektion ohne Kontrastmittelaufnahme	Prävertebrale Flüssigkeitsansammlung von C1–C7	NSAR für 12 Tage	12
*Langford et al. *[6]	Case report	60	m	Nackenschmerzen, reduzierte RoM, Odynophagie, Halsschmerzen	WBC: 13,0 Gpt/l CRP: 4,4 mg/dl	1,1 × 5,6 cm große Raumforderung, als retropharyngealer Abszess gewertet	Prävertebraler Abszess	1. i.v.-Antibiose bzgl. retropharyngealem Abszess 2. Verlegung in ein Zentrum 3. Revision der Diagnose 4. Ketorolac i.v. und nach Entlassung 50 mg Diclofenac 50 mg p.o. 3‑mal/Tag für 10 Tage	10
*Langner et al. *[2]	Case series (*n* = 5)	55,4	3 m, 2 w	Nackenschmerzen (5), Odynophagie (2), reduzierte RoM (5)	WBC: 10,3 Gpt/l CRP: 3,7 mg/dl	Unspezifische Befunde (3)	Prävertebrale Weichteilschwellung (5), prävertebrale Kalzifikation (4)	NSAR (2) Antibiose (4) Kortikosteroide (2) Operative Intervention bei V. a. retropharyngealen Abszess (1)	n. a.
*Joshi et al. *[4]	Case report	46	m	Starke Nackenschmerzen, Kopfschmerzen, Odynophagie	Gering erhöhtes CRP + BSG	n. a.	Retropharyngeale Flüssigkeitsansammlung und Entzündung	Prednisolon 30 mg 3‑mal/Tag + Diazepam 10 mg 2‑mal/Tag + Akupunktur	7
*Kim et al. *[5]	Case series (*n* = 8)	44,5	5 m, 3 w	Nackenschmerzen (8), Odynophagie (7), reduzierte RoM (8), Kopfschmerzen (2)	WBC: 9,9 Gpt/l CRP 3,1 mg/dl	Kalzifikation anterior des Dens axis bzw. Atlas (8)	n. a.	NSAR (8) weiche Halskrause (8) Antibiose (5) Kortikosteroide (1)	5,7

Bei Fallserien werden die Mittlerwerte angeben, in Klammern ist die absolute Anzahl der betroffenen Patienten angegeben

*w* weiblich, *WBC* „white blood count“, *CRP* C‑reaktives Protein, *BSG* Blutkörperchensenkungsgeschwindigkeit, *CT* Computertomographie, *MRT* Magnetresonanztherapie, *d* Tage, *m* männlich, *RoM* „Range of motion“, *NSAR* Nicht steroidale Antirheumatika

Bei Vorliegen von Kontraindikationen von NSAR wurde in der Literatur auch ein Therapieschema von Benzodiazepinen, Kortikosteroiden und Akupunktur vorgeschlagen [4].

Nichtsdestotrotz handelt es sich bei der retropharyngealen Tendinitis um eine zumeist selbstlimitierende Erkrankung, und die Symptome lassen nach ca. 7 bis 10 Tagen nach.

## Fazit für die Praxis

- Der Symptomenkomplex aus atraumatischen, starken Nackenschmerzen, deutlich reduzierter RoM in der HWS, Meningismus und Odynophagie bei erhöhten Infektparametern sollte den Behandler u. a. an eine retropharyngeale Tendinitis denken lassen.
- Eine retropharyngeale Flüssigkeitsansammlung und Kalzifikation im Bereich der oberen Zügel des M. longus colli ist pathognomonisch für eine retropharyngeale Tendinitis.
- Diagnostischer Goldstandard ist die MRT mit Kontrastmittelgabe.
- Therapeutisch bietet sich die hochdosierte NSAR-Therapie, ggf. in Kombination mit Kortikosteroiden p.o., an.
- Die retropharyngeale Tendinitis ist ein aseptisches Krankheitsbild und zumeist innerhalb von ca. 14 Tagen selbstlimitierend.

## Abkürzungen

CRP: C‑reaktives Protein
CT: Computertomographie
ED: „Emergency department“
HADD: „Hydroxyapatite deposition disease“
MRT: Magnetresonanztomographie
NSAR: Nichtsteroidale Antirheumatika
PCR: Polymerasekettenreaktion
PPI: Protonenpumpeninhibitor
RoM: „Range of motion“
RS-Virus: Humanes Respiratorisches Synzytial-Virus
VAS: Visuelle Analogskala
WBC: „White blood count“
